# When do we have the power to detect biological interactions in spatial point patterns?

**DOI:** 10.1111/1365-2745.13080

**Published:** 2018-10-23

**Authors:** Tuomas Rajala, Sofia Charlotta Olhede, David John Murrell

**Affiliations:** ^1^ Department of Statistical Science University College London London UK; ^2^ Centre for Biodiversity and Environment Research University College London London UK

**Keywords:** community ecology, determinants of plant community diversity and structure, interspecific interactions, neighbourhood analysis, null model, spatial point patterns, statistical power

## Abstract

1. Uncovering the roles of biotic interactions in assembling and maintaining species‐rich communities remains a major challenge in ecology. In plant communities, interactions between individuals of different species are expected to generate positive or negative spatial interspecific associations over short distances. Recent studies using individual‐based point pattern datasets have concluded that (a) detectable interspecific interactions are generally rare, but (b) are most common in communities with fewer species; and (c) the most abundant species tend to have the highest frequency of interactions. However, it is unclear how the detection of spatial interactions may change with the abundances of each species, or the scale and intensity of interactions. We ask if statistical power is sufficient to explain all three key results.
2. We use a simple two‐species model, assuming no habitat associations, and where the abundances, scale and intensity of interactions are controlled to simulate point pattern data. In combination with an approximation to the variance of the spatial summary statistics that we sample, we investigate the power of current spatial point pattern methods to correctly reject the null model of pairwise species independence.
3. We show the power to detect interactions is positively related to both the abundances of the species tested, and the intensity and scale of interactions, but negatively related to imbalance in abundances. Differences in detection power in combination with the abundance distributions found in natural communities are sufficient to explain all the three key empirical results, even if all pairwise interactions are identical. Critically, many hundreds of individuals of both species may be required to detect even intense interactions, implying current abundance thresholds for including species in the analyses are too low.
4. *Sy* *n* *thesis.* The widespread failure to reject the null model of spatial interspecific independence could be due to low power of the tests rather than any key biological process. Since we do not model habitat associations, our results represent a first step in quantifying sample sizes required to make strong statements about the role of biotic interactions in diverse plant communities. However, power should be factored into analyses and considered when designing empirical studies.

## 1 INTRODUCTION

Understanding the contribution of biological interactions to the assembly and regulation of natural communities remains a key goal in ecology. The continual development and refinement of methods to detect interactions from spatial, temporal, and spatio‐temporal data have therefore been a mainstay of the literature on the subject.

A particular focus on the role of competition can be found in plant ecology, not least because plants seem to require the same few nutrients (Silvertown, 2004), but also because their sessile nature might permit an understanding of processes from the spatial pattern of individuals (Murrell, Purves, & Law, 2001), and allow for easier experimental manipulation (Goldberg & Barton, 1992). Multiple methods exist to detect interspecific interactions but in non‐manipulative field conditions there are often only two choices, both of which rely upon data where the location, identity, and often size of every individual is recorded (Wiegand et al., 2017). The first option is to fit growth and/or survival models that take into account the identity and size of nearby neighbours (e.g. Comita, Muller‐Landau, Aguilar, & Hubbell, 2010; Stoll, Murrell, & Newbery, 2015; Stoll & Newbery, 2005; Uriarte, Condit, Canham, & Hubbell, 2004). However, this requires repeated sampling over time in order to track the fate of every individual and very often such data are not available. Another issue is that because all interaction parameters are fitted at once, considering all pairwise interactions is very difficult due the large number of parameters. As a consequence neighbouring individuals are sometimes lumped into conspecifics and heterospecifics with the potential problem that interspecific interactions are “lost” due to cancelling out of weak and strong, and/or positive, and negative effects of different species. An extension that has been recently explored is to model survival/growth as functions of the phylogenetic or functional similarity of neighbours (Fortunel, Valencia, Wright, Garwood, & Kraft, 2016; Uriarte et al., 2010). The second option is to investigate the spatial pattern of the community to test the null hypothesis that species are independently arranged with respect to one another. Inference from a single snapshot of the community relies upon the assumption that spatial data retain the “memory” of the birth, death and growth of the individuals (Flügge, Olhede, & Murrell, 2012), and consequently the effect of interspecific interactions should show up as inter‐species spatial dependence after any effect of the abiotic environment has been removed (Murrell et al., 2001). Under the assumption that all pairwise tests are independent, each pair of species can be assessed individually, and dependent interactions are categorised as being a competitive interaction if the species are spatially segregated, and facilitative if they are positively associated in space, although confirmation via experimental manipulation is still advisable. Due to less restrictive data requirements (the test can be carried out on a single sampling of the community), the spatial snapshot option has proven to be very popular, and the test methods employ well‐established spatial statistics such as Ripley's *K* or the pair correlation function (pcf) to test the null model of spatial independence (Wiegand et al., 2012).

The results of previous spatial analyses of multi‐species communities have found only a very low frequency of interspecific spatial interactions (aggregation/segregation) over scales relevant to plant competition, implying interspecific interactions are generally rare, or weak (as discussed by Chacón‐Labella, Cruz, & Escudero, 2017; Luo, Yu, Chen, Wu, & Ding, 2012; Wang et al., 2014; Wiegand et al., 2012). However, comparisons of different plant communities have also revealed a positive relationship between the frequency of spatial independence and the number of species in the community (Chacón‐Labella et al., 2017; Luo et al., 2012; Perry, Miller, Enright, & Lamont, 2014; Wang et al., 2014; Wiegand et al., 2012). Spatial independence between all pairs of species is one of the key assumptions of several unifying theories for biodiversity (McGill, 2010), and the low frequency of detected interactions has been put forward in support of this assertion (Chac′on‐Labella et al., 2017; Perry et al., 2014; Wiegand et al., 2012). However, classical niche theory also predicts the strength of interspecific interactions to decline as the number of coexisting species increases (equation 4 in Chesson, 2000), with the relative strength of interspecific interactions being proportional to 1/(*s* − 1) for *s* species. Therefore, the main difference between the theories is that null models for biodiversity assume spatial independence for all communities regardless of species richness, whereas niche theory predicts spatial interactions are likely to be stronger, and therefore more frequently detected in less species‐rich communities. Hence, we argue the spatial analyses appear to better support the predictions of classical niche theory.

However, both the low frequency of interspecies interactions and the relationship between species richness and species interactions could arise due to the ability of the statistical tests to detect significant interactions at the sample sizes being used. Because of the unequal treatment of the null and alternative hypothesis in classical testing, failure to reject the hypothesis of no interaction does not provide concrete proof of a lack of interactions. As pointed out by Wiegand et al. (2012), when species are rare the rate at which two species might co‐occur in space is also very low and the statistical tests used might not be able to detect any interaction, even if it were very strong. If, as is often the case, species‐rich communities have few common and many rare species, then we would expect to detect few significant interactions. Indeed, several investigations have found the frequency of significant spatial associations between species to be positively related to the abundance of both species being considered (Luo et al., 2012; Wang et al., 2014; Wiegand et al., 2012), raising the possibility that interactions can only be detected amongst the most abundant species.

For all tests, a lower limit on the abundances of species to be included in the analyses must normally be set, and this acknowledges there is a limit to our ability to detect even strong interactions in small sample sizes. Previous investigations have used a range of lower abundance thresholds including 100 (Flügge, Olhede, & Murrell, 2014), 70 (Wiegand et al., 2012), 30 (Perry, Miller, Lamont, & Enright, 2017), and even 18 (Chaćon‐Labella et al., 2017) individuals. However, how and why is the lower threshold of individuals selected? What are the limits of our analyses to detect significant interspecific interactions? We are unaware of any study that investigates the statistical power of the tests for spatial independence between pairs of species that are commonly used and consequently there are no guidelines for the lower abundance threshold. As such care is required when interpreting failures to reject the null hypothesis, and we argue it is hard to make strong statements about the relative roles of stochastic‐ and niche‐based processes across different communities until we gain a better understanding of the power of the methods to detect departures from spatial independence. In other words, is spatial independence a good first approximation in species rich plant communities because of diffuse competition leading to weak interactions, or is it because the statistical methods lack the power to detect the interactions for the given sample sizes typically available?

Here, we will elaborate on the statistical power of commonly used methods to detect significant interactions from spatial point pattern data. We shall study this problem by constructing a simple model for generating bivariate patterns where we can directly control the strength of interaction, and by utilising an approximation to the variance of the spatial summary statistic. We will show how the power to detect significant interactions is very much a function of the species’ abundances, the strength of the interaction (normally the variable we are trying to infer, and therefore unknown), and the spatial scale over which the test is performed. Unfortunately, it is not possible to provide definitive sample size criteria since the power also changes with the summary statistic and test method being used. In particular, for simplicity we ignore habitat associations, and as we will discuss, it is hard to tell how much our results will change when using inhomogeneous tests that take this into account. Despite this, our results are a useful first guide to understanding the sample sizes required to detect pairwise interactions. With this caveat in mind, our analyses will suggest previous abundance thresholds for species inclusion are likely too low to detect even very strong interactions in the most species‐rich communities being tested, thus questioning the previously derived conclusion of a lack of dependence between species. Since power can be estimated from Monte Carlo simulations, we hope our results will motivate ecologists to think more about the issue of sample size in future studies and therefore help to resolve the debate over the relative importance of biotic interactions in species‐rich communities.

## 2 MATERIALS AND METHODS

### 2.1 Summary statistics for bivariate interaction

Consider data for two species labelled 1 and 2 given as two sets of locations of individuals $x_{1}=\left\{x_{11},\cdots x_{{1n}_{1}}\right\}$ and $x_{2}=\left\{x_{21},\cdots x_{{2n}_{2}}\right\}$ respectively, where the locations are observed in a well‐defined area. We will call the combined set of points (**x**_1_, **x**_2_), a bivariate point pattern, and refer to the individuals’ locations simply as points. Technical details are left to Supporting Information Appendix A, but in brief we assume that the data generating mechanisms can be described by some processes *X*_1_ and *X*_2_, and the goal of statistical analysis is to draw conclusions about the processes using the observed set (**x**_1_, **x**_2_). We start by assuming that the processes are second‐order stationary, which means there is no underlying heterogeneity in the abiotic environment (e.g. elevation, soil chemistry) that also affects the distributions of the species, and implies that the statistics calculated from the data do not depend on any particular location in the observation window (see Section 4 for extensions). Although ecological communities are rarely well approximated by stationary models, we motivate studying the stationary case as this must be explored first, before any more complex scenarios can be understood.

We will focus our attention on the second‐order statistic commonly known as Ripley's *K* (Ripley, 1979) and its derivative, the pcf; our rationale being that these two summaries are amongst the most popular when characterising joint dependence (Law et al., 2009; Perry, Miller, & Enright, 2006; Velázquez, Marti;ńez, Getzin, Moloney, & Wiegand, 2016). First (as is standard) we define the intensity of a point process *λ* > 0 as the expected number of points per unit area. The cross‐*K* or partial‐*K*, denoted here by *K*_12_(*r*), is a function defined as the expected number of points of species 2 inside a circle of radius *r* placed on a random individual of species 1, scaled with intensity *λ*_2_ to remove dimension and facilitate comparisons. Due to symmetry, it follows that *K*_12_ (*r*) = *K*_21_(*r*). The parameter *r* controls for spatial scale and allows for multi‐scale analysis.

The derivative of *K*_12_ in *r* is denoted by *g*_12_(*r*), and is called the cross‐ or partial‐pcf. The pcf describes the aggregation/segregation of cross‐species point locations at distance *r* where the probability of having a species 1 individual in some small region and a species 2 individual in some small region distance *r* away is relative to *g*_12 _(*r*)*λ*_1_ *λ*_2_. The quantities are scaled so that for independent processes the expectation is *K*_12_ (*r*) = *πr* ^2^ and *g*_12_ (*r*) = 1. The different statistics are used to ask subtly different questions, with *K*_12_ (*r*) testing for species independence *up‐to *distance *r*, and *g*_12_ (*r*) testing for independence *at* distance *r*.

### 2.2 Model generated data for illustration

For better understanding of the power of bivariate point pattern statistics, we develop a simple two‐species model for which the level of cross‐species aggregation/segregation can be controlled directly and explicitly by two parameters that determine the spatial scale and the strength of the interaction. Using this model, we can provide power estimates for different sample sizes and interaction scales and strengths using simulations. The details of the model are provided in Supporting Information Appendix B. Briefly, we assume species 1 is insensitive to the presence of species 2, but that the spatial distribution of species 2 is dependent on the spatial distribution of species 1. Asymmetric interactions are a reasonable starting point given they are thought to be quite common in plant communities (Freckleton & Watkinson, 2002) and theory suggests competitive asymmetry may help maintain diversity in competitive communities (Nattrass, Baigent, & Murrell, 2012). The locations of all *n*_1_ individuals are given by a Poisson process, so species 1 exhibits no intraspecific spatial structure. The *n*_2_ individuals are placed with distribution that depends on the locations of species 1. Importantly the model has

$$g_{12}\left(r\right)=1+bh\left(r\right),$$

where *h*(*r*) = exp[−*r* ^2^/(2*τ* ^2^)] is a decreasing function whose exponential decay is controlled by the parameter *τ* > 0, and has a range (*h* is non‐zero) of approximately 2*τ*.

This function is analogous to the interaction or competition kernels used in spatially explicit birth‐death models (Murrell, 2010; Murrell & Law, 2003). The strength of interspecies’ interaction, as summarised by *g*_12_ (*r*), is controlled by the parameter *b* ≥ −1. If −1 < *b* < 0 the two species exhibit segregation (*g*_12_ < 1), if *b* > 0 the two species exhibit aggregation or clustering (*g*_12_ > 1), and when *b* = 0 the two species are independent. The reader should note that this model is simply a pattern generating process for illustration, rather than a mechanistic model, and we simulate patterns conditional on fixed *n*_1_ and *n*_2_ as we want full control over them (for the unconditional model the abundances are random, like in the birth and death processes, see e.g. Murrell, 2010). Example point patterns showing inter‐species aggregation and segregation can be found in Supporting Information Figure S6 in Appendix B.

### 2.3 Testing bivariate independence

We now turn our attention to the main problem of determining if the processes *X*_1_ and *X*_2_, as observed through the bivariate point pattern (**x**_1_, **x**_2_), are statistically independent. If the processes were independent, then the observed pattern would be a *random super‐position* of the two processes. We will take this as our *independence* or *null hypothesis* which now needs to be tested using the observed data.

To test if the independence hypothesis is compatible with the data, observed values of a chosen test statistic are compared to the distribution of the test statistic under the independence model. We can test either (a) at some specific distance, which we call *pointwise tests* or (b) simultaneously over multiple distances. For both types of tests, the idea is to compute some test statistic *T* ∈ ℝ from the data, and compare it to the values of *T* (its distribution) as if the null hypothesis were true. If the data value is sufficiently extreme, we have reason to reject the null hypothesis.

The true distribution of the test statistic under independence is rarely known in point pattern applications, and needs to be approximated by an empirical distribution derived from simulations under the independence model. This approach is known as Monte Carlo testing (Myllymäki, Mrkvička, Grabarnik, Seijo, & Hahn, 2017). We consider the observation area to be rectangular, in which case the independence simulation consists of randomly shifting pattern 1 (or 2 or both) with a toroidal wrap (Lotwick & Silverman, 1982). This keeps the intra‐species statistics of the patterns intact while “breaking” any interspecies dependencies, and can also be used for inhomogeneous patterns (Cronie & van Lieshout, 2015).

For the purposes of this discussion, we will consider only the simple pointwise testing scenario, for which we can employ an analytical approach using a Gaussian approximation to the distribution corresponding to the random shift simulations. As we will show, the approximation is very useful since it is not only computationally very efficient relative to the MC simulations, but also allows some analytical insight into what affects the power of the tests. The pointwise tests we will study are comparable to simultaneous tests when the best distance to test at is known (see Supporting Information Table S1 in Appendix C). As detailed in Supporting Information Appendix A, we can choose an unbiased estimator ${\hat{K}}_{12}$ for which approximately it holds:

(1) $${\hat{K}}_{12}∼N\left(K_{12}\sigma^{2}\right)\Leftrightarrow T:\;=\frac{{\hat{K}}_{12}-K_{12}}{\sigma }∼N\left(0,1\right),$$

where *K*_12_ is the value under the correct model. Conditional on the observed point counts *n*_1_,* n*_2_, the variance of ${\hat{K}}_{12}\left(r\right)$ can be approximated by

(2) $$\sigma^{2}\approx c_{1}{\left(n_{1}n_{2}\right)}^{-1}\left[\left(n_{1}+n_{2}\right)c_{2}+c_{3}\right],$$

where *c*_1_,* c*_2_, *c*_3_ are constants depending on the distance *r* and the geometry of the observation area (see Supporting Information Appendix A.3). At a short distance, the constants reflect mainly the stochasticity of each point's neighbour count, and when the distance increases the “censoring” of the neighbourhood at the edge of the finite observation window contributes additional uncertainty. The form given in Equation (1) is exact when *X*_1_ and *X*_2_ are distributed according to a homogeneous Poisson process, but as we will see later on in Section 3.1, the approximation works quite well also for weakly internally aggregated/segregated patterns. Under strong internal aggregation, the true variance of *K*_12_ will be higher than the approximation given by (2), but under strong internal segregation the true variance of the cross‐*K* will be lower than given by (2); we refer to Supporting Information Appendix A.3 for further details. Although we focus on *K*_12_, the approach to approximating the distribution is nearly identical for *g*_12_, only the constants are different.

### 2.4 Power of a statistical test

Denoting the null hypothesis of bivariate independence by *H*_0_, the test statistic by *T*, and a confidence level of the test by (1 − *α*) where *α* ∈ (0,1). Recall that *α* is the researcher's fixed accepted margin of making a false positive decision, also known as *type I error*, defined mathematically as

$$\alpha =P\left(T>q_{1-\alpha }H_{0}\;true\right),$$

where *P* is the distribution of *T*, *q*_1−_ *_α_* is the corresponding threshold value for *T* so that if *T* > *q*_1−_ *_α_* is under *H*_0_, then we reject the null hypothesis *H*_0_. The condition refers to *T* being tested. On the other hand, the *power* of a test is the probability of a true positive judgment, that is, the probability of rejection when the hypothesis *H*_0_ does not hold. Consider first the margin of making a false negative judgment,

$$\beta =P\left(T\leq q_{1-\alpha }H_{0}\;not\;true\right),$$

also known as *type II error*. Then the power of the test is defined as

$$\mathrm{power}=\mathrm{power}\left(H_{0}T\alpha \right):\;=1-\beta .$$

Therefore, a test is powerful if it can correctly reject the wrong null model with a high probability.

Consider the idealised situation of testing the cross‐species independence using the pointwise summary $K_{12}=K_{12}\left(\tilde{r}\right)$ for some fixed spatial scale $\tilde{r}$ only. For the test statistic *K*_12_, the null hypothesis *H*_0_: “random superposition” implies $K_{12}=k_{0}={\pi \tilde{r}}^{2}$. Let us now consider the situation that in truth *K*_12_ = *k*_12_ ≠ *k*_0_. Then, if we accept the approximate Gaussianity of the test statistic as shown in the previous section, it follows by elementary manipulations that

(3) $$power\approx 1-\Phi \left(q_{1-\alpha /2}-\frac{k_{12}-k_{0}}{\sigma }\right),$$

where Φ is the cumulative distribution function of the standard normal distribution, with *a*‐quantiles *q_a_* (approx. 1.96 for *α* = 0.05 in the two‐sided test). Notice that the sign of interaction does not matter, meaning that due to symmetry of the Gaussian distribution aggregation is as easy or hard to detect as segregation of similar strength. Also notice how the power is dependent on the variance (*σ* ^2^) of the test statistic used. The smaller the variance, the higher the power, which explains why different unbiased estimators of *K*_12_ have been developed (see e.g. Illian, Penttinen, Stoyan, & Stoyan, 2008) and, while all being correct in the sense of bias, they can lead to different rates of detecting interactions because of different variances.

We can now use the power formula and our approximation for the variance (Equation (1)) to illustrate how to

- compute the power of the test given the point counts *n*_1_, *n*_2_, expected true signal *k*_12_, and the type I error tolerance *α*;
- compute the required point counts given the expected true signal *k*_1__2_, the type I error tolerance *α,* and the type II error tolerance *β* or power.

## 3 RESULTS

**Figure 1:**
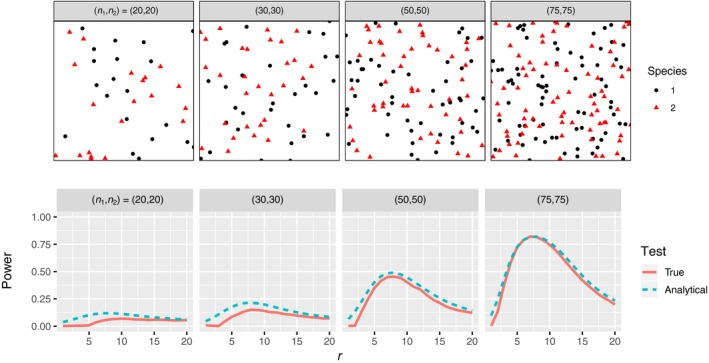
*Top*: Examples of the segregated bivariate point patterns, *b* = −0.5 and 2*τ* = 10, 100 × 100 window. *Bottom*: The power of *K*_12_‐based pointwise cross‐species independence tests when species are segregated like in the example patterns. The true power is estimated using 5,000 repeated tests with 199 random shifts each [Colour figure can be viewed at wileyonlinelibrary.com]

The power formula (Equation (2)) is a good approximation to the power of the toroidal shift Monte Carlo test (Figure 1). There is very little difference between the test's true power and the approximative power given by the analytical formula, with the analytical approximation slightly overestimating the power (at most 10%) due to the clustering of species 2 and subsequent underestimation of the variance via formula (2). The acceptable quality implies that we can discuss the power and its effect on ecological interpretations using the convenient analytical formula, acknowledging the small optimistic bias and the simplifying assumptions of stationarity and weak intra‐species structuring.

As indicated by Equation (1), the variance of the estimator for the *K*_12_‐function is increased when either or both of *n*_1_ and *n*_2_ are small. This means that both the imbalance in population abundances as well as the total number of individuals affect our ability to detect bivariate interactions. We shall investigate each of these in turn, as well as the spatial distance of testing.

### 3.1 Power in balanced scenarios and the importance of the spatial scale of testing

Figure 1 depicts the pointwise powers for different balanced (*n*_1_ = *n*_2_) low‐abundance scenarios when data are segregated (aggregated results are nearly identical). Visual inspection of the example point patterns (Figure 1, top row) already gives some indication that departures from spatial independence might be hard to detect for the lowest abundances. More formal analysis of the power quantifies the increase in ability to detect interactions with increasing abundances (*n*_1_, *n*_2_) of the species being investigated and how this is affected by the spatial scale at which the hypothesis is tested (Figure 1, bottom row). In all cases, the power to detect the interaction at small spatial scales (*r* < 2) is low because, although the interaction is at its strongest here, the variance of *K*_12_ is relatively high and overwhelms the ecological signal. The trade‐off between signal and noise leads to a unimodal relationship between power and the neighbourhood radius *r*, with the peak being approximately at *r* = 7 for the interaction range 2*τ* = 10 for all abundance sizes considered (Figure 1). We will refer to this peak in power with *r* as the optimal distance for testing, and will focus on this best case scenario for the results presented below. The unimodal relationship highlights the point that having some prior knowledge about the likely ranges of biotic interactions is going to be important for detecting interactions.

**Figure 2:**
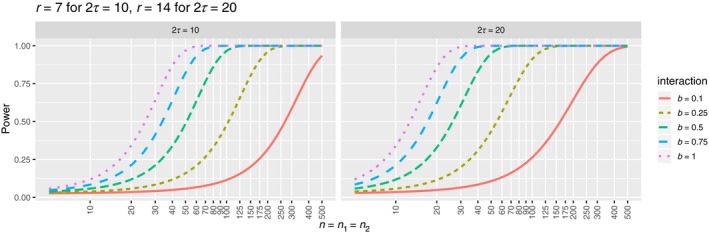
Power of *K*_12_‐based pointwise cross‐species independence tests when abundances are balanced and testing is done on the best possible distance. Test level *α* = 5% [Colour figure can be viewed at wileyonlinelibrary.com]

Previous results based on in situ data analysis suggest detectable interactions between trees typically occur over 10–20 m (Uriarte et al., 2004). Scaling our analyses accordingly, we can use the power formula to estimate the population sizes we require in order to reliably detect an interaction of a given strength and range (Figure 2). If for example, we wish to be 75% sure a true positive is not to be missed when the interaction strength is weak (*b* = −0.1), then we require species with populations of approximately 400 individuals for the 10 unit interaction neighbourhood (2*τ* = 10) and 250 individuals for 20 unit neighbourhood (2*τ* = 20). This value is surprisingly large compared to what data we commonly have available to us.

In contrast, for the maximum possible negative interaction strength (*b* = −1), a similar level of power is reached with only around 35 individuals for 2*τ* = 10 unit and 18 individuals for 2*τ* = 20. Conversely, if we have a pair of species with *n*_1_ = *n*_2_ = 50, and we wish to be 75% sure a true positive is not missed, we must hope that the true interaction *|b| *when coupled with short interaction range (2*τ* = 10) is at least 0.7–0.75, and if coupled with long interaction range (2*τ* = 20) is at least 0.3–0.4. It therefore seems likely that only the very strongest interactions are detectable with the number of individuals that are typically found in the species‐rich datasets.

### 3.2 Imbalances in species abundance

**Figure 3:**
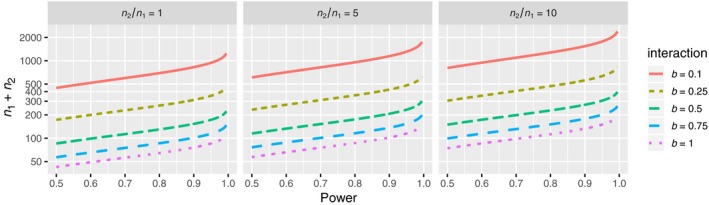
Sample size *n*_1_ +*n*_2_ requirements if testing for independence at level *α* = 5% with a *K*_12_‐based pointwise cross‐species independence test in the example scenario. Interaction range 2*τ* = 10 [Colour figure can be viewed at wileyonlinelibrary.com]

Since most communities exhibit a “hollow curve” distribution of population abundances (McGill et al., 2007), an imbalance in population sizes is very common. From the variance formula (2), it is clear that imbalance has a strong effect on the power because the term (*n*_1_ *n*_2_)^−1^, and hence the variance, increases with imbalance. This relationship is confirmed when we use the power formula to quantify the effect of population imbalance for different interaction strengths and combined population sizes (Figure 3). So, for example, for an interaction strength of |*b*| = 0.1 and a desired power of 80%, a combined individual count of about 750 is required when the populations are perfectly balanced, but 1,000 are required when one species is five times more abundant than the second species, and a surprisingly large 1,500 required when one species is ten times more abundant than the other. Alternatively, consider that we require 90% power, and that the interactions are assumed to be |*b*| = 0.5 and of short range, 2*τ* = 10. Then, to be on the safe side, we should attain samples of sizes at least (100, 100), (40, 200), or (30, 300), depending on the imbalance.

### 3.3 Power at rainforest sample sizes

We now consider how our understanding of the power to detect interactions might affect results for abundance distributions typical of observed plant communities. For simplicity, let us assume interactions are of the type given by our model and that every species is interacting with every other species in an identical manner (so *b* and range 2*τ* are the same for all pairs of species). Since the power is the probability of detecting interactions, given that they exist, we can get a rough estimate of the number of detected cross‐species interactions by assuming the tests are independent, and summing up the powers. This then allows a coarse comparison of recently reported frequencies of detected interactions in tropical forests (Chac′on‐Labella et al., 2017; Lan et al., 2016; Perry et al., 2014; Wang et al., 2014; Wiegand et al., 2012) with the expected frequency of detected interactions as a function of power.

**Figure 4:**
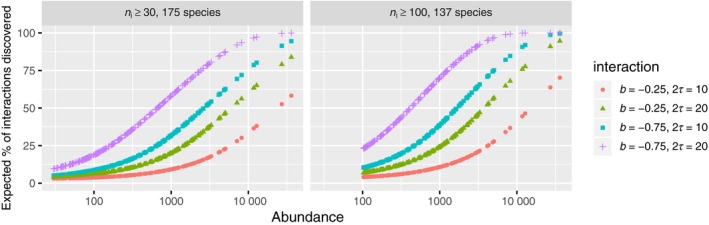
Expected fraction of interaction detected per abundance, if all pairs of species were to interact, and we tested independence with *K*_12_ at optimal distance [Colour figure can be viewed at wileyonlinelibrary.com]

Figure 4 shows the expected number of cross‐species interactions detected as a function of abundance for various hypothetical interaction strengths and ranges. The abundances are taken from the Barro Colorado Island 1995 census (https://ctfs.si.edu/webatlas/datasets/bci/abundance) of woody plants with diameter at breast height at least 1 cm (Condit, 1998), and these are used in conjunction with our variance approximation and bivariate interaction model (so we are not using the spatial point pattern associated with the 1995 census). The abundances are highly skewed, with a large proportion of low‐abundance species, and we show the power in two cases, when the pool of species consists of those with abundance at least 30 and 100. Reducing the species pool by increasing the abundance threshold naturally increases the proportions of detection, and highlights the importance of using similar thresholds when comparing different communities. It is striking how little power is to be expected for most of the species even when assuming strong interaction (*b* = −0.75). Only when the abundance of a species reaches thousands, can we be expected to detect even 50% of the interactions present. This is a very thought‐provoking result, as the lack of detection might be explained simply by a lack of power in the majority of species‐pairs. However, we remind the reader that our tests do not take habitat associations into account whereas the previous analyses (Chaćon‐Labella et al., 2017; Lan et al., 2016; Perry et al., 2014; Wang et al., 2014; Wiegand et al., 2012) approximately factor this out, and being different tests the power to detect interactions will be different. We discuss this point in more detail below.

## 4 DISCUSSION

Understanding the relative strength of interspecific interactions is one of the key goals of community ecology, and the null model approach has been popular for characterising spatial point patterns of (predominantly) diverse plant communities (e.g. Chacón‐Labella et al., 2017; Lan et al., 2016; Martinez, Wiegand, Gonzalez‐Taboada, & Obeso, 2010; Perry et al., 2014; Velázquez, Paine, May, & Wiegand, 2015; Wang et al., 2014; Wiegand et al., 2012; Wiegand et al., 2017). However, there has been little guidance on when a given test is likely to be able to detect species associations that are present. Here, we have made a first step in closing this important gap in our understanding. Our results clarify the quantitative relationships between the strength of the underlying biological interaction, sample size (number of individuals of both species under investigation), and the spatial scale over which the test is being performed. We have also shown that statistical power may explain both the low detection rate of biological interactions in plant communities, and the negative relationship between species‐richness and frequency of detected interspecific interactions in comparative studies.

Ecologists have had to rely largely upon their intuition for deciding the minimum population size to include in their analyses with the result that a range of criteria up to 100 individuals (Flügge et al., 2014) have been used. For species‐rich communities, where many interspecific interactions may necessarily be weak (Chesson, 2000), abundances of both species may need to be in the hundreds of individuals before any interaction is detected (Figure 3), and this implies previous abundance thresholds are likely too low to detect many interactions. As several authors have acknowledged, the failure to reject the null hypothesis of spatial independence in so many species‐pairs does not necessarily mean interspecific interactions are not occurring, or present (Chacón‐Labella et al., 2017; Perry et al., 2014; Wiegand et al., 2012), and we hope our study highlights how the power of the tests can be assessed and should be factored into the interpretation of the results. The power formula can also be used in estimating the area of observation necessary to increase the power to a desirable level (Supporting Information Appendix C.4), so can also be used to aid study design. Nonetheless, we do stress that there is still much to be learned about the power of the statistical tests used in earlier studies, given the assumptions we had to make, and that the reader should take our contribution as a guide to sample sizes that are required to make strong statements about the frequency and strength of interspecific interactions.

Although our model is clearly mis‐specified as we use tests assuming that intensity is not dependent on abiotic features of the environment, the general applicability of our results will carry‐over into the inhomogeneous setting. The quantitative relationships between sample sizes and statistical power will of course change for different tests, including the inhomogeneous Poisson process, but the qualitative relationships we uncover are likely to remain. In particular, we would still expect a positive relationship between population size and frequency of interactions to emerge simply due to an increase in power at larger sample sizes. Such a positive relationship has already been reported in a number of empirical studies that take habitat associations into consideration (Chacón‐Labella et al., 2017; Luo et al., 2012; Wang et al., 2014; Wiegand et al., 2012). It is possible that common species are better competitors and are somehow suppressing the abundance of the weaker competitors, but without experimental manipulation, or perhaps different analyses using repeated sampling over time (Damgaard & Weiner, 2017), it is hard to distinguish whether this pattern is a result of biological processes or the ability of the statistical methods to detect interactions at different population sizes. Given the potential for low power to detect interactions for abundances typical of species‐rich communities, we suggest that future tests should consider the neighbourhood from the perspective of traits or phylogenetic relatedness (e.g. Wang et al., 2016), with interactions potentially being some function of relatedness or functional similarity. In so doing, tests would consider the impact of multiple species on a focal species and would reduce some of the low power issues we highlight here. However, such an approach requires reliable phylogenies, and adequate sampling of traits that are relevant to growth, survival and fecundity of individuals, and these are still challenging issues in ecology.

The spatial scale over which tests are performed is important for the ability to detect spatial dependencies (Figure 1), and our results are similar to empirical studies that often find few negative interactions at the shortest distances, even though this is where the interactions are likely to be strongest (Chacón‐Labella et al., 2017; Wang et al., 2014; Wiegand et al., 2012). Short scales suffer from having high variability due to the relatively small number of neighbours possible in a small area, but at longer distances, the effect of neighbours is weaker. Hence, there is a sweet spot where this trade‐off is maximised, and the location of this is likely dependent on several factors, not least of which is the scale over which interactions are occurring (see e.g. figure 2 in Chacón‐Labella et al., 2017 for an empirical example). For woody plants, there have been several studies that have fitted neighbourhood growth or survival models to individual‐based data that track the fate of trees over time (e.g. Stoll & Newbery, 2005; Uriarte et al., 2004), and most results seem to point to interactions being confined to 10–30 m radius around an individual. However, little is known about how the spatial scales of interspecific interactions change with life history stage, environmental conditions, or even species identity even though the latter has been shown to be very important for determining coexistence (Murrell & Law, 2003). Any changes to the scales of interactions will have consequences for the hypothesis testing methods discussed here, but until more is understood about the spatial scales of interactions between species, it seems sensible to test over ranges reported in earlier studies.

Our discussion up to this point has been in the context of stationary, most notably homogeneous, data. Most recent analyses have tried to factor out the effects of spatial heterogeneity in the abiotic environment by using inhomogeneous Poisson processes as the null model (Chacón‐Labella et al., 2017; Punchi‐Manage et al., 2015; Wiegand et al., 2012). Currently, it is hard to predict whether the power of an inhomogeneous analogue of our scenario would be lower or higher. On the one hand, we could expect higher power to detect interactions because the model better captures the underlying processes that generate the spatial distributions of the species within the community. However, we also expect variance to be increased, since extra parameters need to be estimated leaving fewer degrees of freedom per parameter. For example, tests using the inhomogeneous Poisson process method use a smoothing kernel to approximately remove the effects of large scale structure assumed to be caused by habitat associations (see e.g. Wiegand et al., 2012). Typically, the same smoothing parameter is used for all species, which is a sensible assumption when little is known about the spatial scale of habitat associations, but there is no reason to suspect a single smoothing parameter is appropriate for all species. An open challenge is to better understand how mis‐specification of the smoothing parameter will bias the detection of interactions. Again, we feel that using a biologically motivated model to simulate data is a useful approach for exploring such issues.

The understanding of statistical significance versus the practical importance of any effect has been discussed in other application domains (Button et al., 2013; Hojat & Xu, 2004; Sawyer & Ball, 1981). The difficulties inherent in studies determining significance in settings with relatively small sample sizes, has for example been noted by Ioannidis (2005) and Open Science Collaboration et al. (2015). Our results underscore the difficulties of statistical testing for smaller sample sizes, especially given the unequal weighting between the null and alternate hypotheses. We also remind the reader that the spatial statistics used in the null model approach do not say anything directly about the processes that may have created the patterns, and different processes could generate the same summary statistic. Since the data are often a single snapshot of the community it is also hard to infer the importance of the results for the population dynamics of the species under scrutiny. For example, other processes such as temporal variation in the environment (Chisholm et al., 2014), and within species interactions (perhaps mediated via specialist natural enemies, LaManna et al., 2017) could both contribute more to population dynamics than any detected pairwise interaction. Similarly, interactions that are undetected due to low sample sizes could be expected to contribute little to population dynamics, especially if both species are quite rare, although the contribution of many weak interactions might still be significant factors affecting growth, survival, and/or fecundity. As a result, we believe the spatial tests for independence should act as exploratory studies to highlight potentially significant species interactions, but understanding their biological importance requires different methods. As an alternative, model‐based approaches, either in the form we use here (which include the familiar Thomas Cluster models) or birth‐death models (May, Huth, & Wiegand, 2015; Rajala, Murrell, & Olhede, 2018) could also be applied to the inference of biological interactions from point pattern data (Wiegand et al., 2017). Model fitting will normally lead to estimation of parameters that can also be estimated in the field (e.g. dispersal kernels, interaction kernels), we therefore feel that their continued development will help to improve the understanding of the processes underpinning the results returned (Wiegand et al., 2017).

In conclusion, we hope our main contribution is to encourage more users to consider explicitly the ability of the spatial point pattern tests to detect significant associations between species. We have shown that the data requirements to detect even strong interactions may be quite high, mirroring results for null model tests of species co‐occurrences in community matrix data (Freilich, Wieters, Broitman, Marquet, & Navarrete, 2018; Gotelli, 2000). On this basis, we suggest it is desirable to only interpret the frequency of interactions across large numbers of species once the effect of different powers to detect interactions for pairs of species of given population sizes has been (even approximately) factored out. This seems especially important in comparative analyses across different communities where the spatial scales, strengths of interactions, and the species abundance distributions may differ and affect the power to detect interactions.

## AUTHORS’ CONTRIBUTIONS

T.R. conceived the idea during discussions with D.J.M. and S.O.; T.R. derived the model and formulas, designed and executed computations, and contributed extensively to the manuscript; D.J.M. contributed to the rainforest experiment and extensively to the manuscript; S.O. contributed to the manuscript. All the authors contributed to the intellectual core of the manuscript.

## Data Availability

The abundances in Section 3.3 for the Barro Colorado Island 1995 census are available at http://ctfs.si.edu/webatlas/datasets/bci/abundance/.
