# Supplementary material for: When do we have the power to detect biological interactions in spatial point patterns?
Source: J Ecol. 2018 Oct 23;107(2):711–21. doi: 10.1111/1365-2745.13080 (PMC6472561; doi:10.1111/1365-2745.13080)
Supplement: Supplementary file 1 [file JEC-107-711-s001.pdf]

Supplements for the paper "When do we have the power to detect biological interactions in spatial point patterns?" by Rajala, Olhede and Murrell.

A Technical details on point processes and summaries

A.1 Preliminaries

We observe two point processes X_1 and X_2, observed as point patterns, or equivalently as sets of locations, $\mathbf{x}_1 = \{x_{11}, ..., x_{1n_1}\}$ and $\mathbf{x}_2 = \{x_{21}, ..., x_{2n_2}\}$ in a finite observation window $W \subset \mathbb{R}^2$. Write $N_i(B) := \#(X_i \cap B)$ for the random number of points of type i in a set $B \subset \mathbb{R}^2$. All results generalise easily to higher dimensions. We assume that the processes (X_1, X_2) are jointly second order stationary, so that the expectation of the statistics we shall calculate do not depend on any particular location in the observation window.

A.2 Summary statistics for bivariate interaction

First assumption is that the expected point count in any set B can be written as an integral

$$\mathbb{E}N_i(B) = \int_B \lambda_i(u)du,$$

where $\lambda_i(u) \geq 0$ is called the intensity. For stationary processes $\lambda_i(u) \equiv \lambda_i$ is a constant, and we assume that $\lambda_i > 0$, so that $\mathbb{E}N_i(B) = \lambda_i|B|$.

We define the cross-K function as a function of a distance parameter $r > 0$

$$K_{12}(r) := \lambda_2^{-1}\mathbb{E}_{o1}N_2(b(o,r)),$$

where $b(o,r)$ is a ball of radius $r > 0$ centred at the origin, the expectation $\mathbb{E}_{o1}$ is conditional on the joint process having a point of type 1 at the origin o (for stationary processes the exact location does not matter). Heuristically, $\lambda_2 K_{12}(r)$ is the mean abundance of species 2 within distance r of a typical point of species 1. Equivalently we can define $K_{21}(r)$, but due to symmetry $K_{12} = K_{21}$.

The cross-K index is a powerful statistic for testing purposes, but for a more detailed description of spatial interactions we often study the derivative of K_{12},

$$g_{12}(r) := \frac{K_{12}'(r)}{2\pi r},$$

known as the cross (or partial) pair correlation function (pcf). The pcf describes the aggregation/segregation of cross species point locations: The probability of having a species 1 point at some small region dx and a species 2 point at some small region dy is given by $g_{12}(||x - y||)\lambda_1\lambda_2 dxdy$. If the processes are independent, $g_{12}(r) \equiv 1$ and $K_{12}(r) = \pi r^2$. We say the processes are aggregated if $g_{12} > 1$, and segregated if $g_{12} < 1$, at any particular distance $r > 0$.

To estimate these quantities several estimators have been proposed, differing in how the observation bias near the borders of W is corrected. We will look at bivariate

versions of the globally corrected "Ohser"-type estimators (Illian et al. 2008, p. 230, Ward and Ferrandino (1999) and Wiegand et al. 2016) of the form

$$c(r)\frac{1}{n_1n_2}\sum_{x\in\mathbf{x}_1}\sum_{y\in\mathbf{x}_2}f_r(x-y)=c(r)T(r)$$

where $c(r)$ is some constant, mainly responsible for scaling and edge correction and possibly depending on r, and f_r is some function, for example an indicator function for K_{12} and a kernel function for g_{12}. Note that while the theoretical K_{12} and g_{12} are symmetric in the species indices, their estimators are not necessarily so (see e.g. Lotwick and Silverman 1982).

A.3 Covariance of the summary statistics

The key ideas for this Section follow those given by Lotwick and Silverman 1982. Consider two point processes X_1 and X_2 with fixed counts n_1, n_2 in a bounded window W. We are interested in the covariance between different distances $r > 0, s > 0$ of estimators of type

$$M(r)=c(r)\frac{1}{n_1n_2}\sum_{x\in X_1}\sum_{y\in X_2}f_r(x-y)=c(r)T(r),$$

where f_r is symmetric in $x-y$ (this can be extended to non-symmetric functions). Then, if X_1 and X_2 are independent,

$$\begin{aligned}Cov[T(r),T(s)]=(n_1n_2)^{-2}[\,&n_1(n_1-1)n_2(n_2-1)a_1(r,s)+n_1n_2(n_1-1)a_2(r,s)\\&+\;n_1n_2(n_2-1)a_3(r,s)+a_4(r,s)-(n_1n_2)^2a_5(r,s)]\end{aligned}$$

where

$$\begin{aligned}a_1(r,s)&=\mathbb{E}f_r(x-y)f_s(x'-y')\\a_2(r,s)&=\mathbb{E}f_r(x-y)f_s(x'-y)\\a_3(r,s)&=\mathbb{E}f_r(x-y)f_s(x-y')\\a_4(r,s)&=\mathbb{E}f_r(x-y)f_s(x-y)\\a_5(r,s)&=\mathbb{E}f_r(x-y)\mathbb{E}f_s(x'-y'),\end{aligned}$$

with expectations over random locations on W such that the pair x, x' follow the distribution of X_1 and the pair y, y' follow the distribution of X_2. Heuristically, for the K_{12} which effectively counts point pairs, the terms reflect the probabilities of all different types of point pair occurrences, and then get multiplied by corresponding number of possible combinations. For example, a_1 counts the occurrences of two separate pairs which both have a point from each species, and a_2 and a_3 count the times an individual has two neighbours both of the opposite species. These terms are all influenced by the edge of the observation window W, and are therefore sensitive to the size of the neighbourhood radii r, s. In the case of i.i.d. uniform locations (that is, assuming X_1 and X_2 follow Poisson

processes and we condition on fixed n_1 and n_2), we have $a_1 = a_5$ and $a_2 = a_3$, and the covariance, say $Cov_0(r,s)$, simplifies to

$$\begin{aligned} Cov_0(r,s) &= (n_1 n_2)^{-1}[\ (n_1+n_2-2)a_2(r,s) + a_4(r,s) - (n_1+n_2-1)a_1(r,s)] \\ &= (n_1 n_2)^{-1}[\ (n_1+n_2)c_2(r,s) + c_3(r,s)] \end{aligned}$$

where we have further simplified using

$$\begin{aligned} c_2 &= a_2(r,s) - a_1(r,s) \\ c_3 &= a_4(r,s) + a_1(r,s) - a_2(r,s) \end{aligned}$$

to highlight the effect of sample sizes. The additional constant for the statistic M is $c_1(r,s) = c(r)c(s)$, which mainly scales away biases due to edge effects. This is the form we provide in Equation 2 and use for our examples.

For the general situation where the processes have some pair correlation functions g_1 and g_2, additional terms appear such that

$$\begin{aligned} Cov[T(r),T(s)] = Cov_0(r,s) &+ \\ &(n_1 n_2)^{-1}[\ (n_1-1)(n_2-1)e_1(r,s) + (n_1-1)e_2(r,s) + (n_2-1)e_3(r,s)] \\ &\approx Cov_0(r,s) + e_1(r,s) + n_2^{-1}e_2(r,s) + n_1^{-1}e_3(r,s) \end{aligned}$$

with

$$\begin{aligned} e_1(r,s) &= |W|^{-4}\int_{W^4} \big\{[g_1(x-x')-1][g_2(y-y')-1] + [g_1(x-x')-1] \\ &\qquad + [g_2(y-y')-1]\big\} f_r(x-y) f_s(x'-y') dx dy dx' dy' \\ e_2(r,s) &= |W|^{-3}\int_{W^3} [g_1(x-x')-1] f_r(x-y) f_s(x'-y) dx dy dx' \\ e_3(r,s) &= |W|^{-3}\int_{W^3} [g_2(y-y')-1] f_r(x-y) f_s(x-y') dx dy dy'. \end{aligned}$$

From these expression we can see that if the marginal processes tend towards internal clustering, i.e. $g_1 \geq 1$ and $g_2 \geq 1$ the extra terms will be positive and the covariances will be higher than with just uniformly random marginals. This means that in our examples where the species 2 is slightly clustered (cf. Section B), our plug-in variances are slightly lower than in truth, leading to over-estimation of power. This explains the "optimistic" bias we see in e.g. Figures 1 and S8. Also noteworthy is that for processes tending towards internal segregation ($g_1 \leq 1, g_2 \leq 1$) the extra terms are negative and the covariances and variances are lower than for uniformly random marginals. From a statistical point of view the best patterns to analyse are strongly internally segregated.

The evaluation of $Cov_0(r,s)$ is not trivial, as all the terms are geometrical integrals determined by the weighting function f and the observation window W. For estimator of the pair correlation function g_{12}, consider the box-kernel

$$f_r(x-y) = (2h)^{-1}1_{b_r}(x-y),$$

where $b_r = b(o, r+h) \setminus b(o, r-h)$ and $h > 0$ is the bandwidth. Then

$$\begin{aligned} a_1(r,s) &= |W|^{-4}(2h)^{-2}\left[I_1(r+h) - I_1(r-h)\right]\left[I_1(s+h) - I_1(s-h)\right] \\ a_2(r,s) &= |W|^{-3}(2h)^{-2} I_3(r,s) \\ a_4(r,s) &= |W|^{-2}(2h)^{-2} I_2(r,s,h), \end{aligned}$$

where

$$\begin{aligned} I_1(r) &= \int_{b(o,r)} |W \cap (W+z)| dz \\ I_2(r,s,h) &= I_1(r+h) - I_1(s-h) \quad \text{if } |r-s| < 2h, \quad \text{and 0 otherwise} \\ I_3(r,s) &= \int_W \int_W \int_W 1_{b_r}(x-y) 1_{b_s}(x-z) dx dy dz. \end{aligned}$$

For the K_{12} function we use $f_r(x-y) = 1_{B_r}(x-y)$ where $B_r = b(o,r)$, and

$$\begin{aligned} a_1(r,s) &= |W|^{-4} I_1(r) I_1(s) \\ a_2(r,s) &= |W|^{-3} I_4(r,s) \\ a_4(r,s) &= |W|^{-2} I_1(\min(r,s)) \end{aligned}$$

where

$$I_4 = \int_W \int_W \int_W 1_{B_r}(x-y) 1_{B_s}(x-z) dx dy dz.$$

The quantities I_3 and I_4 can be approximated numerically using Monte Carlo integration, and I_1 is up to a constant the isotropised set covariance of W which has a closed form solution for some elementary shapes of W (rectangle, disc; Illian et al., 2008, p. 485).

A.4 Gaussian approximation of $\hat{K}_{12}$

The mathematics of the limiting behaviour of the "Ohser"-type estimators are beyond this study, and for progress in this regard we refer to Heinrich (2015). We resort to the same argument as Wiegand et al. (2016): the empirical plots do not show signs against normality apart from very short distances due to the positivity constraint. Fig. S5 illustrates this (compare to Wiegand et al. 2016, Fig. S3). Note the accuracy of the analytical formula derived in Appendix A.3 for the variance.

B Model generated data

The process is inspired by the shot-noise product Cox processes (Jalilian et al., 2015), and is constructed hierarchically. First, let X_1 be a stationary Poisson process with intensity λ_1. Then conditional on a realisation $\mathbf{x}_1$ of X_1, let X_2 be an inhomogeneous Poisson process with intensity function

$$\lambda_2(u; \mathbf{x}_1) = e^a \prod_{x \in \mathbf{x}_1} (1 + bh(u-x)) \in \quad u \in W$$

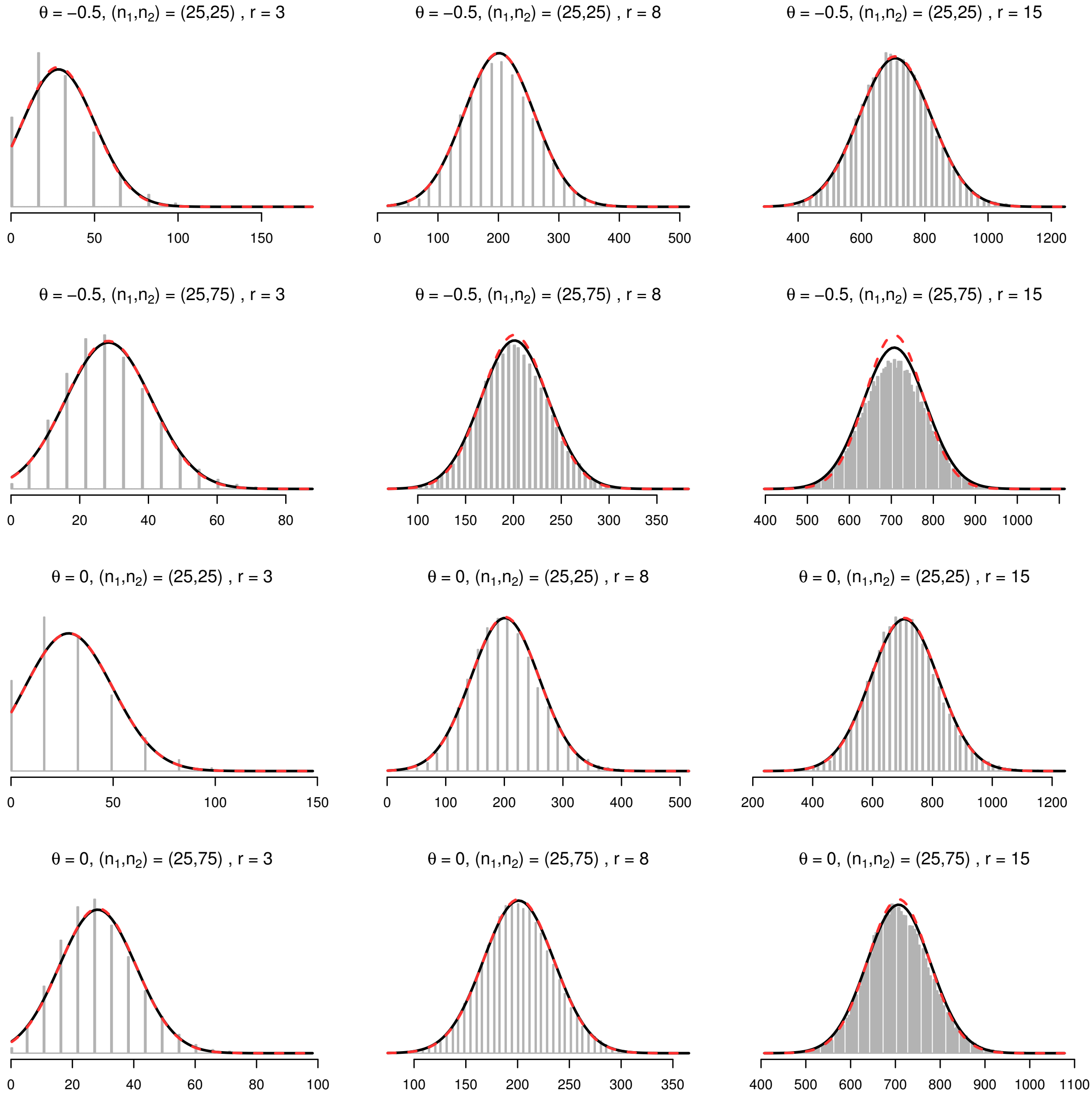

Figure S5: Empirical distribution of $\hat{K}_{12}$ at $r = 3, 8, 15$ for random shifted data, where data is generated by the introduced bivariate model with range $2\tau = 10$. Overlaid are Gaussian density functions with empirical mean and variance (solid, black) and with theoretical mean πr^2 and variance σ^2 given by the analytical approximation (dashed, red), which in most subplots is superimposed with the solid black line.

where $h(v) = k_\tau(v)/k_\tau(0)$ with k_τ a 2D kernel function (probability density) with standard deviation $\tau > 0$, and $a \in \mathbb{R}, b > -1$ are parameters controlling the intensity and the interaction, respectively. The joint model is stationary, and isotropic if k is isotropic, and has

$$\lambda_2 = \exp\left(a + \lambda_1 b/k_\tau(0)\right), \quad g_{11}(u) \equiv 1, \quad g_{12}(u) = 1+bh(u), \quad g_{22}(u) = \exp\left(\lambda_1 b^2 (h * h)(u)\right)$$

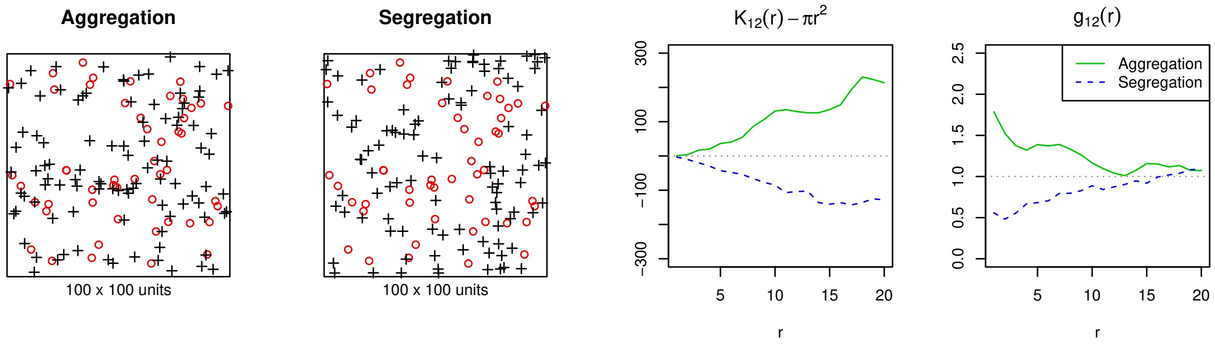

Figure S6: Example bivariate point patterns exhibiting cross-species spatial aggregation and segregation, and the corresponding cross-K and cross-pcf statistics.

where $*$ denotes convolution. From these properties we see that if $-1 < b < 0$ the two species exhibit segregation ($g_{12} < 1$), and if $b > 0$ the two species exhibit aggregation or clustering ($g_{12} > 1$), and when $b = 0$ the two species are independent. We also see that the both types of interactions result in clustering of species 2 ($g_{22} \geq 1$). The range of interaction (if defined via the pair correlation) is controlled by the parameter τ. In our examples we use a Gaussian kernel, for which the range, i.e. h is non-zero, is approximately 2τ. Fig. S6 shows two examples of the process with identical type 1 patterns, together with their K_{12} and g_{12} estimates.

C Additional power estimates

Fig. S7 provides evidence that the analytical power formula is close to the true power, which can be estimated by Monte Carlo simulation, also in unbalanced scenarios. Compare Fig. S7 to Fig. 1.

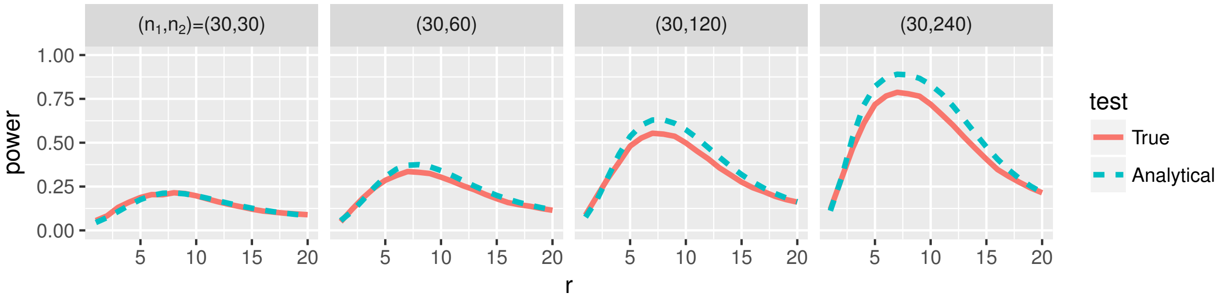

Figure S7: Power of K_{12}-based pointwise cross-species independence tests with varying degrees of imbalance $n_1 = 30 \leq n_2$. Range of interaction $2\tau = 10$. The true power is estimated using 5000 repeated tests with 199 random shifts each.

Fig. S8 provides evidence that the analytical power formula works also for the cross-pair correlation function g_{12}. Compare Fig. S8 to Fig. 1. The optimistic bias in the power is again due to the slight downwards bias in the variance as species 2 is clustered. The estimation was carried out in this example with the bandwidths $0.15\sqrt{\frac{|W|}{n_1}}$ as the samples sizes were balanced.

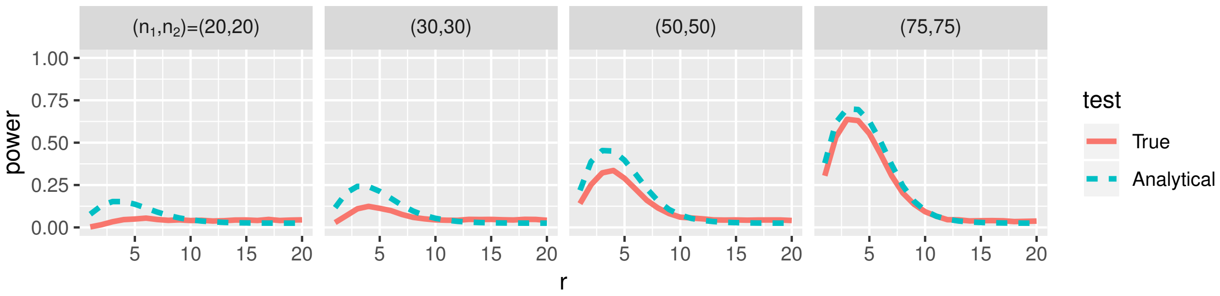

Figure S8: Power of g_{12}-based pointwise cross-species independence tests. Range of interaction $2\tau = 10$. The true power is estimated using 5000 repeated tests with 199 random shifts each.

C.1 Testing without simulation

Note that the approximate Gaussianity and the approximate variance formula lead directly to a χ^2-test of independence without random shift simulations, much like in the work by Wiegand et al. (2016). Procedure:

1. Estimate $\hat{K}_{12}(r)$ for one r
2. Compute $\sigma(r)$
3. Compute $T = (\hat{K}_{12}(r) - \pi r^2)^2/\sigma^2(r)$
4. Compare T to the χ^2-distribution with 1 degrees of freedom.

C.2 Pointwise test vs testing over a range

In the simulation experiments we control all factors, so we can choose the distance of the pointwise test to be optimal, i.e. the distance which we know the power is highest. Table S1 compares this optimal pointwise power to the power of a test where instead of a single distance an interval of distances is tested simultaneously using a deviation test (see e.g. Myllymäki et al., 2017).

	(n_1, n_2)=(25,25)			(n_1, n_2)=(50,50)			(n_1, n_2)=(75,75)		
Interaction model	r=1-10	r=1-20	pw.o.	r=1-10	r=1-20	pw.o.	r=1-10	r=1-20	pw.o.
$b = -.25, 2\tau = 10$	0.11	0.05	0.07	0.29	0.14	0.16	0.50	0.28	0.30
$b = -.25, 2\tau = 20$	0.18	0.10	0.15	0.41	0.43	0.43	0.70	0.78	0.73
$b = -.75, 2\tau = 10$	0.43	0.21	0.31	0.96	0.82	0.82	1.00	1.00	0.99
$b = -.75, 2\tau = 20$	0.71	0.77	0.80	1.00	1.00	1.00	1.00	1.00	1.00

Table S1: Power comparison of the Studentised L^2 deviation test over two distance intervals ("r=1-10" and "r=1-20") with the pointwise power formula at the known optimal distance ("pw.o."). The K_{12} statistic, and the deviation test powers estimated using 1000 simulations per model and/or setting as indicated, with 199 random shifts each.

We tried using the covariance formula to combine several distances to a χ^2-test, but the very short distance asymmetry and the non-central χ^2 did not immediately lead to a useful power approximation of the Studentised L^2 test.

C.3 Improving power by combining summaries

A simple way to improve power is to combine several summaries in the test statistic. As an example, we combined the K_{12} with the nearest neighbour distance distribution function D_{12} (Van Lieshout and Baddeley, 1999) by using the pointwise test statistic

$$T_{KD}(r) = \left(\frac{\hat{K}_{12}(r) - \bar{K}_{12}(r)}{\hat{\sigma}_K(r)}\right)^2 + \left(\frac{\hat{D}_{12}(r) - \bar{D}_{12}(r)}{\hat{\sigma}_D(r)}\right)^2.$$

Fig. S9 depicts the pointwise powers for K_{12}, D_{12} and the combination when the data was generated by our bivariate model with $b = 0.5$, $2\tau = 10$. The nearest neighbour summary operates only at short distances as it saturates to 1 quickly, and for distances > 5 is inferior to K_{12} in this scenario. But as it captures different information than the K_{12}, combining it with K_{12} increases the power, at least when $r < 10$. After $r > 10$ the combined pointwise power is diminished as the nearest neighbour summary provides no help yet is weighted equally with K_{12} in making the decision. Weighting the statistics by their useful ranges is therefore recommended.

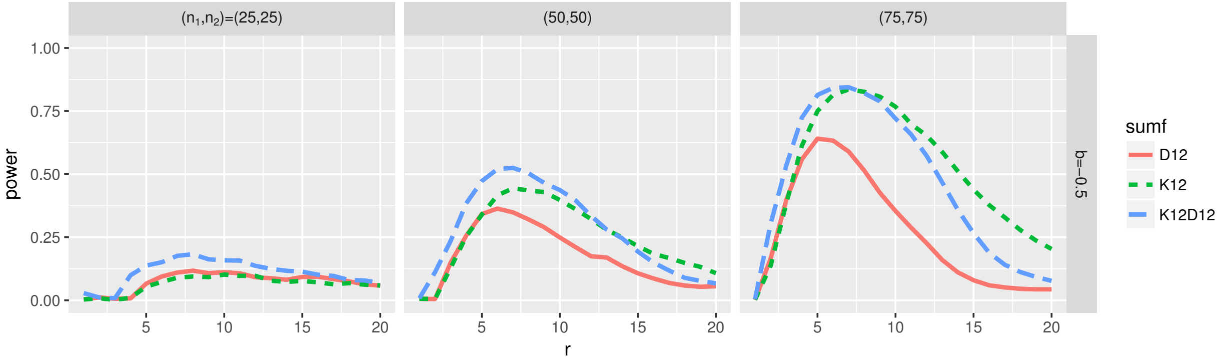

Figure S9: Power of K_{12}, D_{12} and $K_{12} + D_{12}$-based pointwise cross-species independence tests when species are significantly segregated.

Table S2 gives the powers of a test with $T = \sum_{r=1}^{20} T_{KD}^2(r)$, over the distances $1 - 20$. The power with the combined statistic is higher than with either of the components alone for small samples.

$(n_1, n_2) =$	(25,25)	(50,50)	(75,75)
D_{12}	0.09	0.27	0.45
K_{12}	0.08	0.43	0.83
$K_{12} + D_{12}$	0.14	0.49	0.82

Table S2: Power of the independence test when using K_{12}, D_{12} or both, for different balanced sample size, deviation test over distances 1-20. Each power was estimated using 2000 simulations of data, 199 random shifts each.

C.4 Sample size and observation window

Under most circumstances samples sizes can only really be increased by increasing the area of observation, and the connection $\lambda_i \approx n_i/Area$ can be used to get a rough idea of the requirements. First, a pilot study needs to be conducted to estimate λ_i (see Illian et al. 2008 for estimation techniques). Then we need to determine the minimum $Area$, accounting for imbalance between species if that is needed. Table S3 gives some example calculations when a square area is used (note that the window geometry might affect the power; see C.5).

Balance λ_2/λ_1	1	2	5	10	50
Area requirement, $\lambda_1 \approx 0.01$	130^2	110^2	90^2	77^2	55^2
Area requirement, $\lambda_1 \approx 0.5$	18^2	16^2	13^2	11^2	8^2

Table S3: Required observation area given estimates of intensities λ_1 and λ_2, when expected interaction has strength $b = 0.25$ and range $2\tau = 10$, testing with $K_{12}(r = 7)$ at level $\alpha = 5\%$ and requiring power at least 90%.

C.5 Additional factors

The geometry of the area has an effect on the estimator's variance and hence the power of the test, but according to the analytical formula the effect is relatively small. For example, if we change from a square shape to an elongated rectangle shape with equal area but width-to-height -ratio 3, and consider interaction $b = 0.25$ and type I error level $\alpha = 5\%$, the power drops from 33.2% to 32.9% with $2\tau = 10$ and 78.0% to 76.8% with $2\tau = 20$ for sample size $(80, 80)$, and from 15.4% to 15.3% with $2\tau = 10$ and 41.4% to 40.5% with $2\tau = 20$ for sample size $(30, 80)$.

Increasing the type I error level α increases the power as illustrated in Table S4. From the table we can see that a 5% increase in α can reduce the type II error $\beta = 1 - power$ by more than 10%. So in scenarios where we can tolerate some extra false positive discoveries with the simultaneous decrease in false negatives, for example when pre-screening a large data set for more involved downstream analysis on found interacting pairs, adjustments to α should be considered.

(n_1, n_2) =	(10,10)	(30,30)	(50,50)	(80,80)	(30,50)	(30,80)
α =1%	0.01	0.08	0.26	0.68	0.14	0.24
α =5%	0.06	0.21	0.48	0.86	0.32	0.47
α =10%	0.10	0.31	0.61	0.92	0.44	0.59

Table S4: Power of the K_{12} independence test at most powerful distance for typical type I error levels α. Interaction $b = 0.5$, $2\tau = 10$.

Appendix References

L. Heinrich. Gaussian limits of empirical multiparameter K-functions of homogeneous Poisson processes and tests for complete spatial randomness. *Lithuanian Mathematical Journal*, 55(1):72–90, 2015.

J. Illian, A. Penttinen, H. Stoyan, and D. Stoyan. *Statistical analysis and modelling of spatial point patterns*. Wiley & Sons, 2008.

A. Jalilian, Y. Guan, J. Mateu, and R. Waagepetersen. Multivariate Product-Shot-noise Cox point process models. *Biometrics*, 71(4), 2015.

H. W. Lotwick and B. W. Silverman. Methods for analysing spatial process of several types of points. *Journal of the Royal statistical Society*, 44(3):406–413, 1982.

M. N. M. Van Lieshout and A.J. Baddeley. Indices of Dependence Between Types in Multivariate Point Patterns. *Scandinavian Journal of Statistics*, 26(4):511–532, dec 1999.

J.S. Ward and F.J. Ferrandino. New derivation reduces bias and increases power of Ripley's L index. *Ecological Modelling*, 116(2-3):225–236, 1999.

T. Wiegand, P. Grabarnik, and D. Stoyan. Envelope tests for spatial point patterns with and without simulation. *Ecosphere*, 7(June):1–18, 2016.
